# Human follicular fluid shows diverse metabolic profiles at different follicle developmental stages

**DOI:** 10.1186/s12958-020-00631-x

**Published:** 2020-07-23

**Authors:** Jihong Yang, Ting Feng, Suying Li, Xinyue Zhang, Yun Qian

**Affiliations:** Reproductive Medicine Center of the Second Affiliated Hospital of Nanjing Medical University, Nanjing, 210011 China

**Keywords:** Metabolic profiles, Follicular fluid, Follicle, Follicular development

## Abstract

**Background:**

To investigate the metabolic profiles in the follicular fluid (FF) samples from patients undergoing in vitro fertilization (IVF) and to analyze the correlations with follicular development.

**Methods:**

The FF samples were obtained from participants (*N* = 26) who were receiving IVF under the gonadotropin-releasing hormone agonist (GnRH-a) long protocol stimulation and were collected separately from small (8–13 mm) and large (17–22 mm) follicles at the time of oocyte retrieval. Metabolomic analysis of the FF samples was performed using liquid chromatography-tandem mass spectrometry (LC-MS/MS).

**Results:**

The results demonstrated that the size of the follicle influences the metabolic signature of the FF according to the profile and differential metabolites. Dehydroepiandrosterone (DHEA), which is enriched in steroid hormone biosynthesis, correlated negatively with the oocyte maturation rate and the high-quality embryo rate, and thus could be used to estimate the predictive diagnostic potential of follicular development.

**Conclusion:**

The FF has different metabolic characteristics in different stages of follicular development. Exploring meaningful metabolites could predict follicular development, and modifications of these metabolites could influence follicular development.

## Background

Metabolomics comprehensively investigates all metabolic fingerprints, which can reflect the current state of organisms directly and accurately, thereby helping to understand biological processes and mechanisms more effectively. Metabolites, as the downstream products in system biology, influence cellular metabolism and reflect the overall outcome of biological events [1]. Scholars have used metabolomics to reveal the physiological state of organisms and to identify possible biomarkers for fertility research [2]. Castiglione Morelli et al. found that metabolic profiling could be applied to identify biomarkers to diagnose polycystic ovary syndrome (PCOS) and endometriosis, and could be used to predict the developmental potential of oocytes and subsequent pregnancy outcome [3].

As the immediate and necessary microenvironment of oocytes, follicular fluid (FF) provides nutrients and growth factors that promote oocyte growth, development, and viability by accumulating products from all types of metabolism [4, 5]. A study found that changes in the constituents of the FF, such as phthalate, might affect the steroidogenic capacity or viability of follicular cells, and thereby alter the levels of ovarian hormones [6]. In addition, Marei et al. pointed out that high levels of palmitic acid in the FF were associated with a negative pregnancy outcome after intracytoplasmic sperm injection (ICSI) [7]. Thus, the FF composition is significantly affected by metabolic factors, and the analyses of metabolites in FF could reveal the correlation between metabolites and oocyte quality, embryo development and pregnancy outcome.

Oocyte maturation is associated with follicle size; therefore, the size of the follicle on the day of oocyte retrieval is one of the determinants of oocyte maturation [8]. Research has shown that the oocyte recovery rate from small follicles (8–12 mm) at oocyte retrieval is significantly lower than that of medium (13–23 mm) and large (≥ 24 mm) follicles [9], which means that the larger the follicle, the higher the oocyte recovery rate, and therefore the greater the chance of mature oocytes being obtained. However, it has not been determined whether the metabolite components in the FF at different developmental stages from the same background are different. Therefore, we used a common metabolomics research method, non-targeted metabolomics [10], to analyze metabolic alterations in the FF from follicles of different sizes to discover the effect of metabolites on follicular development and oocyte maturation in a uniform background. Meanwhile, it is clinically significant to use metabolomics to explore molecular targets with diagnostic or predictive value for follicular development, for use as clinical indicators of infertility and IVF outcome.

## Materials and methods

### Study population

This study was a prospective clinical trial. A total of 26 participants who underwent IVF from Apr to Nov 2019 at the Reproductive Medicine Center of the Second Affiliated Hospital of Nanjing Medical University, China, were recruited into this study. We included women whose infertility was caused by tubal factors or unexplained infertility and their male spouses could provide normal sperm for IVF to control the effects of male compromising factors on the results. Unexplained infertility in this study was defined as meeting the criteria of infertility and no successful pregnancy after two rounds of intrauterine insemination. We believe that follicular development in these women was normal and there was no influence on follicular development that would reduce errors compared with other included populations. The exclusion criteria were patients with a history of endometriosis, PCOS, premature ovarian failure, ovarian surgery, or other factors. All participants provided written informed consent. The FF was collected from the women following the first cycle of ovarian stimulation in an agonist protocol. The Ethics Committee of the Second Affiliated Hospital of Nanjing Medical University reviewed and approved this study. This study was registered in the China clinical trial registration center under the registration number ChiCTR1900022319.

### Ovarian stimulation protocol

The ovarian stimulation protocol was as follows. At the middle luteal phase of the previous menstrual cycle, gonadotropin-releasing hormone agonist (GnRH-a) (triptorelin, Lizhu Pharmaceutical Trading Co., China) was injected subcutaneously at a dose of 0.1 mg/day. Vaginal ultrasound and blood tests were performed on days 2–4 of the menstrual cycle to monitor the antral follicle count (AFC) and basal sex hormones. Once the examination results met the pituitary suppression criteria, the participants would be injected intramuscularly with gonadotropin (Gn) at 150–225 IU/day. Meanwhile, the dose of GnRH-a was reduced to 0.05 mg/day until the day of human chorionic gonadotropin (HCG, Lizhu Pharmaceutical Trading Co., China) administration when one or more follicles reached a diameter of 18 mm and the estradiol (E2) concentrations were appropriately elevated and matched the number of follicles [11]. Thereafter, 10,000 IU HCG was administered and oocyte retrieval was performed 34–36 h later.

### FF sample collection

At 34–36 h after intramuscular injection of HCG, oocytes and their corresponding FF were retrieved using transvaginal ultrasound-guided aspiration. To collect the FF from a single individual follicle, in one participant, a small (average follicular diameter was 8–13 mm, corresponding to the FF volume of 0.3–1.0 ml) follicle was first aspirated independently using a fresh needle [12]. The oocyte was collected in a dish with the appropriate culture media, and the total volume of the FF was placed in a 15 ml sterile test tube. After puncture of the first ovary, the needle was removed and flushed, and then the air was aspirated until the tubing was empty. Then, the first aspiration of a large (average follicular diameter was 17–22 mm, corresponding to the FF volume of 2.5–5 ml) follicle in the second ovary was initiated. The volumes of the collected FF were recorded and subsequently correlated to the diameter of the corresponding follicles, as measured before oocyte retrieval by transvaginal ultrasonography. If the volume of the FF did not match the corresponding follicle size, the sample would not be included in the study. The FF samples from small and large follicles were centrifuged at 10000 × *g* for 10 min, and the supernatants were collected and stored at − 80 °C for subsequent analysis.

### The process of oocyte fertilization and assessment of embryo development

The oocytes were removed from the follicles and maintained in the culture media (6% CO2, 37 °C) until insemination occurred 4 h later. After 4 h, the sperm and cumulus oophorus around the oocyte were mechanical removed by repeated aspiration. Concurrently, the maturity of oocytes were assessed, whereby oocytes at GV (germinal vesicle nucleus) or MI (metaphase I oocytes) stage were considered immature, whereas those at MII (metaphase II oocytes, 1st polar body) stage were deemed mature. If unfertilized oocytes were present, remedial ICSI would be performed and the sample was excluded. Fertilization, cleavage and quality embryos were observed under a stereoscope at 24, 28 and 72 h after fertilization. The fertilized oocytes were then transferred to the global total LP for further culture, a culture of human embryos from zygote to blastocyst (LifeGlobal, H5GT-200507C, USA). The grading for the quality of the embryos was as follows: Grade I - day-3 embryo with uniform blastomeres and fewer than 5% fragments; Grade II - day-3 embryo with uniform blastomeres and 5–20% fragments; Grade III - day-3 embryo with nonuniform blastomeres and 20–50% fragments; Grade IV - day-3 embryo with nonuniform blastomeres and more than 50% fragments. High-quality embryos were referred to as Grade I and Grade II.

### Untargeted metabolomics analysis

Metabolomic analysis was performed by Wuhan Metware Biotechnology Co., Ltd., (Wuhan, 430,070, China). Briefly, 300 μl of methanol was added to the FF sample (100 μl), and the mixture was homogenized in an ice-water bath for 0.5 h. The sample was subsequently centrifuged at 12,000×*g* for 10 min at 4 °C, and the supernatant (200 μl) was transferred to a centrifuge tube. After incubating at − 20 °C for 0.5 h, the supernatant was centrifuged at 12,000 r/m for 15 min at 4 °C. The recovered supernatant was used for liquid chromatography-tandem mass spectrometry (LC-MS/MS) analysis. The samples were analyzed using an LC-electrospray ionization (ESI)-Tandem mass spectrometry system.

The LC-MS/MS analysis was conducted using a Waters ACQUITY UPLC HSS T3 C18 column with a multistep gradient (mobile phase A of 0.01% formic acid in ultra-pure water and mobile phase B of acetonitrile) at a flow rate of 0.3 ml/min over a run time of 14 min.

The mass spectrometer was operated in ESI positive and negative modes with voltages of 250 or 1500 V. The gas temperature and sheath temperature were both set at 325 °C, with a gas flow rate of 8 ml/min. The quality control samples were prepared from a mixture of sample extracts and were used to analyze the repeatability of samples under the same treatment method. During the instrumental analysis, a quality control (QC) sample was inserted into every 15 FF samples to monitor the repeatability of the analysis process.

### Statistical analyses

The obtained LC-MS/MS data files were first extracted using the Profinder software for information such as the mass to charge ratio, retention time, and peak area of the characteristic peak, and then the peak alignment and statistical analyses were conducted using Mass Profiler Professional software (Agilent Technologies, Santa Clara, CA, USA). The selected peak indices, with accurate m/z and fragment information, were submitted to an online library search, including Metlin and Kyoto Encyclopedia of Genes and Genomes (KEGG). Univariate statistical analyses included Student’s t-test and difference multiple analysis, and multivariate statistical analyses consisted of principal component analysis (PCA), and orthogonal partial least squares discriminant analysis (OPLS-DA). Multivariate statistics were performed using the Metaboanalyst 4.0 software, while Student’s t-test and the correlation analysis with clinical data were performed using SPSS version 13.0 (IBM Corp., Armonk, NY, USA). The heatmap was constructed using MeV software. In addition, MetPA software was used for enrichment analysis of KEGG pathways. Finally, Stata was used to perform receiver operator characteristic (ROC) curve analysis and to calculate the area under the curve (AUC), to estimate the predictive diagnostic potential of follicular development by using metabolomic biomarkers in FF. The results are presented as the mean ± the standard error of the mean (SEM), and *p* < 0.05 was considered statistically significant.

## Results

### Clinical epidemiological information

The relevant clinical epidemiological information for the 26 participants and their male spouses is shown in Table 1 and Supplemental Table 1. The mean age of the subjects in this study was 29.50 ± 0.62 years, with a body mass index (BMI) of 21.21 ± 0.38 kg/m^2^, basal serum follicle-stimulating hormone (FSH) of 8.24 ± 0.69 mIU/ml, and Anti-Müllerian Hormone (AMH) of 4.18 ± 0.49 ng/ml. The number of oocytes retrieved was 13.27 ± 1.24 and the oocyte maturation rate was 77.05 ± 3.35 (%). These parameters were all in the normal range.

**Table 1:** Clinical epidemiological information for participants who provided the FF sample

Parameters	Values
Age (year)	29.50 ± 0.62
BMI	21.21 ± 0.38
Percentage of infertility type (%)	
Primary infertility	57.69
Secondary infertility	42.31
Basal E2 (pg/ml)	50.99 ± 4.48
Basal LH (mIU/ml)	4.51 ± 0.33
Basal FSH (mIU/ml)	8.24 ± 0.69
AMH (ng/ml)	4.18 ± 0.49
AFC	15.38 ± 1.01
Gn dose (IU)	2336.54 ± 107.90
Gn duration (days)	9.81 ± 0.36
E2 level on the trigger day (pmol/L)	4136.54 ± 353.29
Progesterone level on the trigger day (nmol/L)	0.98 ± 0.10
LH level on the trigger day (IU/L)	1.09 ± 0.12
No. of oocytes retrieved	13.27 ± 1.24
Oocyte maturation rate (%)	77.05 ± 3.35
Fertilization rate (%)	78.42 ± 3.02
Cleavage rate (%)	98.84 ± 2.30
High-quality embryo rate (%)	31.53 ± 4.83
Biochemical pregnancy rate (%)	65.33 ± 8.90
Clinical pregnancy rate (%)	53.33 ± 9.39

Data are presented as mean values and standard error

For one participant, oocyte maturation rate = number of oocytes at MII (metaphase II oocytes, 1st polar body) stage/ total number of oocytes retrieved

Fertilization rate = number of fertilized embryos/total number of oocytes retrieved

Cleavage rate = number of cleavage embryos/number of 2PN

High-quality embryo rate = number of high-quality embryos (grade I and II embryos)/number of 2PN

Biochemical pregnancy rate refers to the cumulative biochemical pregnancy rate in the oocyte cycle during which the samples were collected, that is, the number of biochemical pregnancies of the participant/the number of embryos transplanted in this cycle

Clinical pregnancy rate refers to the cumulative clinical pregnancy rate in the oocyte cycle during which the samples were collected, that is, the number of clinical pregnancies of the participant/the number of embryos transplanted in this cycle

On the day of oocyte retrieval, the follicle sizes matched the FF volumes. The small follicles were 11.43 ± 0.25 mm and their corresponding FF volumes were 0.73 ± 0.04 ml. The large follicles were 19.43 ± 0.20 mm and the corresponding FF volumes were 3.72 ± 0.10 ml.

### Metabolic differences in the FF from small and large follicles

**Fig. 1:**
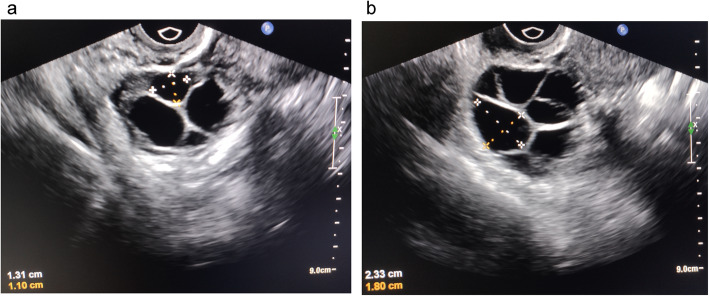
Different sizes of follicles in one woman were selected. **a** Small follicles, with an average follicular diameter of 8–13 mm, corresponding to the FF volumes 0.3–1.0 ml. **b** Large follicles, with an average follicular diameter 17–22 mm, corresponding to the FF volumes 2.5–5 ml

**Fig. 2:**
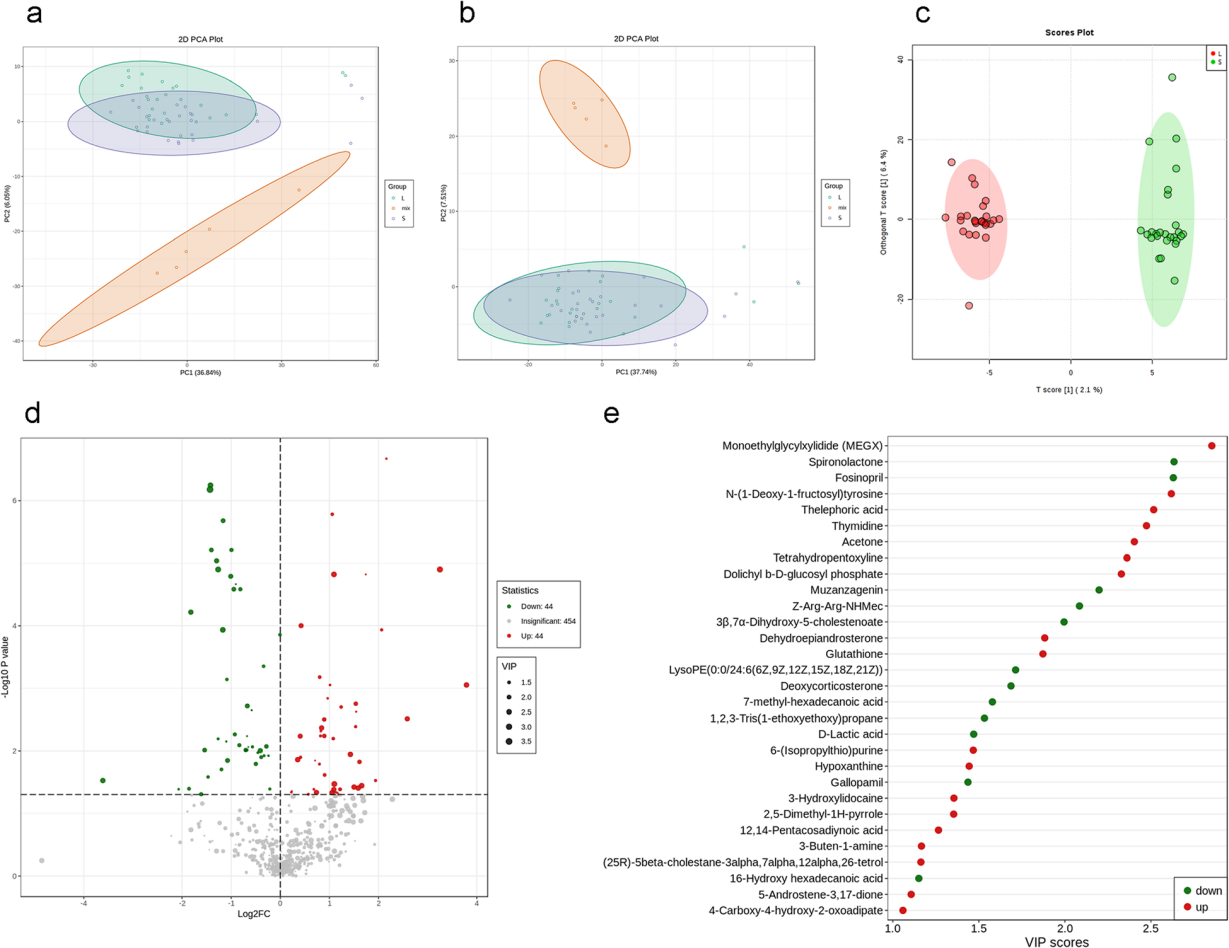
Principal component analysis (PCA) plots and two-dimensional (2D) clustering plots describing the trend of separation between 26 independent samples between two groups. The Large and Small groups are represented by green and purple circles, respectively. The red circles are the QC samples. **a** The plots of PCA score in the ESI positive ion mode. **b** The plots of PCA score in the ESI negative ion mode. **c** OPLS-DA score plot demonstrating separation between the two groups in ESI positive and negative ion modes, based on the regression model. The OPLS-DA shows a prediction power (Q2) of 0.489 and a correlation index (R2Y) of 0.972, according to cross-validation. Samples from the Large group are indicated by red nodes; the samples from the Small group are indicated by green nodes. **d** Volcano plots presenting the statistical significance of metabolites in the FF samples in ESI positive and negative ion modes. **e** Change trends of 30 differential metabolites; the horizontal axis shows the VIP score of each substance. A VIP score is a measure that summarizes the contribution of a given variable to the regression model

**Fig. 3:**
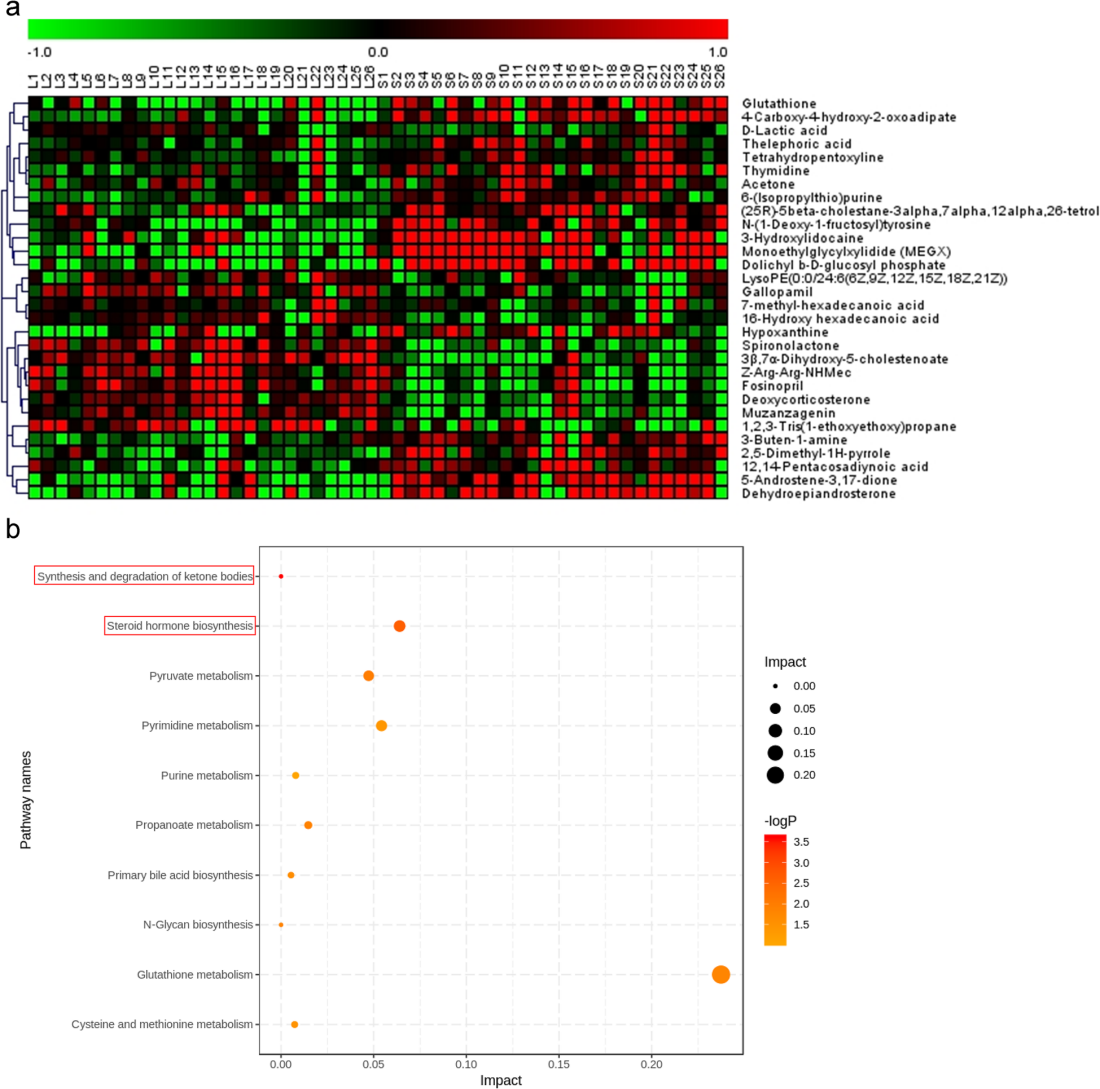
**a** Heatmap plot of upregulation and downregulation for 30 differential metabolites with VIP > 1 and *P* < 0.05 identified by LC-MS/MS. **b** Overview of pathway analysis based on altered metabolisms suggested by MetPA

LC-MS/MS was used to detect metabolites in 52 FF samples, comprising 26 samples from small follicles and 26 samples from large follicles under both ESI positive and negative ion modes (Fig. 1). As shown in Fig. 2a-b and Supplemental Fig. 1a, QC samples clustered together in the PCA, which indicated that the LC-MS/MS system exhibited high stability and reproducibility. The plots of PCA scores showed no obvious separation between the groups of the small and large follicles in both ESI positive and ESI negative ion modes. Subsequently, to better illustrate the metabolic variations in FF, OPLS-DA was applied to process the data, which exhibited good separation (Fig. 2c, Supplemental Fig. 1b-c). The results showed that 980 compound signals were found in the ESI positive mode and 740 in the negative mode. The compound signals with variable importance in projection (VIP) > 1 and *p* < 0.05 were screened, which identified 63 metabolites in the ESI positive mode and 25 metabolites in the negative mode. This result was illustrated using a volcano plot (Fig. 2d, Supplemental Fig. 1d-e). Furthermore, by searching the Metlin database and comparing the compound signals of mass and retention time, 30 metabolites were identified for further analysis (Table 2). The change types of these 30 substances are shown in Fig. 2e and Supplemental Fig. 1f-g. In addition, their corresponding information was also shown in a heatmap, which could intuitively indicate the differences in the metabolites between the two sample groups (Fig. 3a, Supplemental Fig. 2a-b).

**Table 2:** LC-MS/MS detected metabolites that varied in FF with significant difference

Metabolite	RT (min)	m/z	*P* values	Type
LysoPE(0:0/24:6(6Z,9Z,12Z,15Z,18Z,21Z))	9.638	613.3383	0.049	down
Gallopamil	9.255	484.2938	0.026	down
Tetrahydropentoxyline	4.265	366.1415	0.047	up
Thelephoric acid	5.07	352.0188	0.039	up
7-methyl-hexadecanoic acid	9.927	270.255	0.040	down
Glutathione (GSH)	1.589	307.0826	0.015	up
4-Carboxy-4-hydroxy-2-oxoadipate	1.043	280.0444	0.002	up
16-Hydroxy hexadecanoic acid	10.212	272.2331	0.041	down
Thymidine	4.311	242.0889	0.011	up
6-(Isopropylthio)purine	7.79	240.067	0.002	up
Acetone	4.216	58.0418	0.049	up
D-Lactic acid	1.284	90.0322	0.0001	down
1,2,3-Tris(1-ethoxyethoxy)propane	6.567	308.222	3.27E-03	down
Fosinopril	5.874	563.2989	3.40E-09	down
Muzanzagenin	8.594	442.2702	6.28E-07	down
3β,7α-Dihydroxy-5-cholestenoate	8.692	454.3049	1.30E-07	down
Z-Arg-Arg-NHMec	5.902	621.3027	3.15E-08	down
Spironolactone	5.918	433.2275	6.36E-06	down
Deoxycorticosterone (DOC)	8.116	330.2213	1.34E-03	down
3-Buten-1-amine	0.776	71.0736	5.33E-05	up
Hypoxanthine	1.698	136.039	6.86E-04	up
(25R)-5beta-cholestane-3alpha,7alpha,12alpha,26-tetrol	10.786	436.3543	4.33E-04	up
2,5-Dimethyl-1H-pyrrole	0.956	95.0714	3.43E-04	up
5-Androstene-3,17-dione	8.49	286.1953	6.36E-10	up
12,14-Pentacosadiynoic acid	11.285	374.3223	5.70E-04	up
N-(1-Deoxy-1-fructosyl)tyrosine	0.969	360.1529	5.40E-07	up
Dolichyl b-D-glucosyl phosphate	4.878	488.2174	2.39E-04	up
Dehydroepiandrosterone (DHEA)	8.559	288.2111	1.22E-04	up
Monoethylglycylxylidide (MEGX)	4.821	206.1432	3.76E-07	up
3-Hydroxylidocaine	5.37	250.1696	6.60E-06	up

Type: Metabolites in small follicles were compared with those in large follicles, i.e., metabolites in large follicles were used as the control group

**Fig. 4:**
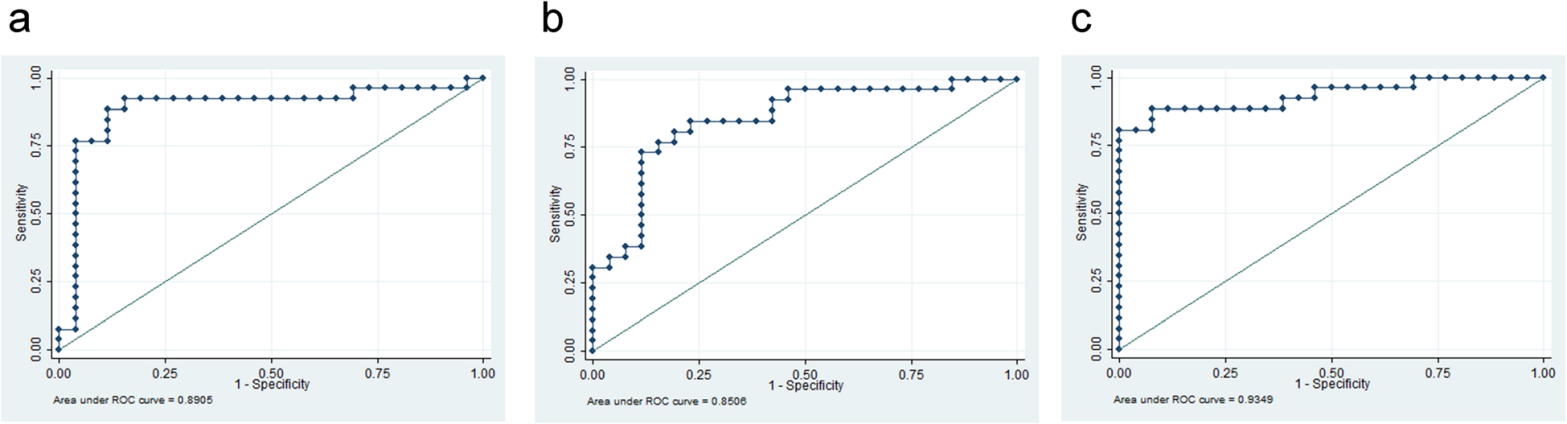
**a** ROC curves of DOC in the FF for follicle size. **b** ROC curves of DHEA in the FF for follicle size. **c** ROC curves of the combination of the above two metabolites for follicle size

KEGG pathway enrichment analysis showed that these 30 metabolites were mainly concentrated in 10 metabolic pathways dominated by synthesis and degradation of ketone bodies (*p* = 0.00994) and steroid hormone biosynthesis (*p* = 0.004891) (Fig. 3b, Supplemental Fig. 2c-d). Acetone, as the main differential metabolite enriched in the synthesis and degradation of ketone bodies pathway, was upregulated in the FF of small follicles compared with that in large follicles. The level of deoxycorticosterone (DOC) was lower, while that of dehydroepiandrosterone (DHEA) was significantly higher, in the FF of small follicles compared with those in the FF from large follicles. DOC and DHEA were enriched in steroid hormone biosynthesis pathway. After statistical consideration, DOC and DHEA were selected from among the metabolites enriched by the KEGG pathways. For DOC, the AUC was 0.8905 with 84.62% sensitivity and 88.46% specificity (Fig. 4a). For DHEA, the AUC was 0.8506 with 84.62% sensitivity and 57.69% specificity (Fig. 4b). When combining the two biomarkers together, AUC was 0.9349 with 88.46% sensitivity and 76.92% specificity (Fig. 4c).

In addition, we matched a total of 1720 metabolites in the FF found under both ESI positive and negative ion modes in the small follicles and large follicles from each participant, and found differences in 17 out of 26 participants (65.38%).

### Correlations between differential metabolites and clinical epidemiological information

The FF of the small and large follicles originated from the same participant; therefore, we selected their ratios (the ratio of metabolites in small follicles to those in large follicles, in other words, metabolites from large follicles were used as the control group and small follicles as the experimental group) for the 30 differential metabolites and general clinical indicators for comparison. Different correlation patterns were found for different clinical indicators. The correlation coefficients and their corresponding *p*-values are shown in Table 3. Among them, the evaluation index of ovarian function status, such as the basal follicle-stimulating hormone/luteinizing hormone (FSH/LH), correlated positively with thymidine and negatively with 4-carboxy-4-hydroxy-2-oxoadipate. AMH correlated positively with thymidine and DHEA. Three metabolites, gallopamil, hypoxanthine, and 4-carboxy-4-hydroxy-2-oxoadipate, correlated negatively with the progesterone level on the trigger day, whereas thymidine correlated positively. Medication related indicators (Gn duration and Gn dose) were also investigated. Gn duration and Gn dose both correlated negatively with glutathione (GSH) and DOC, while Gn duration correlated positively with thelephoric acid.

**Table 3:** Pearson correlation coefficients between the statistical significance of metabolites and clinical epidemiological information in small/large FF samples

Metabolites	Small/large FF
**Basal FSH/LH**	
Thymidine	0.576 (0.002)
Gallopamil	−0.589 (0.002)
6-(Isopropylthio)purine	0.502 (0.009)
4-Carboxy-4-hydroxy-2-oxoadipate	− 0.572 (0.002)
**AMH**	
Thymidine	0.469 (0.016)
1,2,3-Tris(1-ethoxyethoxy)propane	−0.400 (0.043)
Muzanzagenin	0.588 (0.002)
Z-Arg-Arg-NHMec	0.615 (0.001)
Dehydroepiandrosterone (DHEA)	0.405 (0.040)
**Progesterone level on the trigger day (nmol/L)**	
Gallopamil	−0.506 (0.008)
Thymidine	0.454 (0.020)
4-Carboxy-4-hydroxy-2-oxoadipate	−0.444 (0.023)
2,5-Dimethyl-1H-pyrrole	0.423 (0.031)
Hypoxanthine	−0.494 (0.010)
**Gn duration (days)**	
Thelephoric acid	0.464 (0.017)
Glutathione (GSH)	−0.470 (0.015)
Deoxycorticosterone (DOC)	−0.421 (0.032)
3-Buten-1-amine	−0.394 (0.047)
**Gn dose (IU)**	
Glutathione (GSH)	−0.474 (0.014)
Deoxycorticosterone (DOC)	−0.538 (0.005)
**Oocyte maturation rate (%)**	
Dehydroepiandrosterone (DHEA)	−0.502 (0.012)
**High-quality embryo rate (%)**	
Dehydroepiandrosterone (DHEA)	−0.487 (0.016)

In parentheses, the Pearson correlation p-value was illustrated if it is less than 0.05

In addition, DHEA correlated negatively with the oocyte maturation rate and the high-quality embryo rate. However, no metabolites were associated with the rate of fertilization and cleavage associated with embryonic development or with clinical outcomes (biochemical and clinical pregnancy rates).

## Discussion

We conducted metabolomic analysis of the FF to explore the links between metabolites and follicle size. The results demonstrated that different stages of follicular development influenced the metabolic signature of the FF, as profiled by LC-MS/MS. By examining the FF of the small and large follicles, 1720 metabolites were detected by ESI positive and negative ion modes. According to their *p*-values and VIP-values, 30 differential metabolites were finally obtained after screening and comparison with the Metlin database. To investigate the metabolic pathways involving these different metabolites from both ESI positive and negative modes, we performed KEGG pathway enrichment analysis. KEGG pathway enrichment analysis found that the 30 metabolites were enriched in 10 metabolic pathways, among which the synthesis and degradation of ketone bodies and steroid hormone biosynthesis showed significant differences. In addition, DOC and DHEA, which were enriched in steroid hormone biosynthesis, could be used to estimate the predictive diagnostic potential of follicular development. We further investigated the potential relationship between metabolites in the FF and the clinical epidemiological information of the participants, and found that DHEA correlated positively with AMH and negatively with the oocyte maturation rate and the high-quality embryo rate. Meanwhile, GSH and DOC correlated negatively with the sensitivity of the body to Gn. This indicted that higher AMH levels resulted in a higher ratio of DHEA, i.e., a higher content in small follicles and a lower content in large follicles, further reducing the oocyte maturation rate and the high-quality embryo rate. However, GSH and DOC levels in small follicles were higher than those in large follicles; therefore, Gn duration and Gn dose were less useful.

Ovaries, especially cumulus cells, secrete a variety of steroid hormones, which affect the development and maturation of oocytes and follicles, and ultimately promote or inhibit ovulation [13, 14]. Studies have shown that steroid hormone biosynthesis is one of key signaling pathways related to reproductive regulation [15, 16].

DHEA is an essential prohormone in ovarian follicular steroidogenesis [17] and can be converted to more active forms such as testosterone, estradiol, and androstenedione [18]. Thus, on the one hand, DHEA is thought to be a key molecule in maintaining the critical balance between androgen and estrogen production, and on the other hand, it affects oocyte maturation. Li and his team found that DHEA in the FF correlated negatively with IVF clinical outcomes, specifically the number of oocytes retrieved, the fertilization rate, the number of viable embryos, and the number of blastocysts produced [19]. Our results showed that the DHEA level in small follicles was increased and correlated negatively with the oocyte maturation rate and the high-quality embryo rate, indicating that DHEA correlates negatively with follicular development, oocyte maturational status and embryo quality. Besides DHEA, the dehydroepiandrosterone sulfate (DHEA-s) level in the FF could influence the oocyte maturation process, and predict fertilization and embryonic development ability [20]. Kushnir et al. found that the lower the ratio of androstenedione to DHEA, the higher the concentration of DHEA, which has been reported to be associated with better IVF prognosis [21]. In our research, androstenedione and DHEA were differential metabolites, although both metabolites were upregulated in small follicles compared with that in large ones. However, the androstenedione/DHEA ratio in small follicles was 0.67 ± 0.18, while in large follicles it was 0.39 ± 0.20, with no statistical difference (*p* = 0.31). There was no significant difference in the association between DHEA and clinical outcome (biochemical and clinical pregnancy rates), possibly because our included population were all participants of reproductive age with normal ovarian function, and the differences between the samples were small.

Pearson correlation analysis showed that AMH correlated positively with DHEA. In women, AMH is secreted by ovarian granulosa cells and is observed in pre-antral and small antral follicles. The level of AMH and AFC were consistent, and could reflect the ovarian reserve [22, 23]. When antral follicles develop and become larger, AMH expression decreases gradually [24]. Thus, the number of small antral follicles increases, when the level of AMH increases, which leads to fewer mature antral follicles, failure to provide the necessary substrates for estrogen generation, and furthermore, to hyperandrogenism (such as high testosterone, DHEA) and ovarian dysfunction [25]. The data and statistical results of the present study are consistent with the views of authors of previous studies, indicating that it is feasible to compare the metabolites between the small and large follicles from the same individual. Meanwhile, medication related indicators (Gn duration and Gn dose) both correlated negatively with GSH and DOC. Gn has a stimulatory effect on GSH [26]; therefore, Celik et al. detected that the concentration of GSH was higher in mature oocytes than in immature oocytes [27]. Thus, Gn might increase the GSH level in oocytes. In addition, DOC can stimulate Gn release, promote the development of follicles, and induce LH surges to ovulate [28]. DOC was discovered to effectively induce GVBD at all concentrations, and regulates the final oocyte maturational stages in fish [29, 30]. These two metabolites are associated with the sensitivity of the body to Gn and could be used as prediction indices for Gn. However, these data are from the FF, and no studies have been done in blood. Thus, further serological research is required.

In the present study, we included all female participants of reproductive age who participated in IVF due to tubal factor or unexplained infertility. Although such inclusion criteria led to a homogeneous background, they also made the results less extensive and narrowed their scope of application. The FF from a small follicle and a large follicle were collected from the same participant; therefore, we compared the metabolites in the FF of these two follicles and found that most of the participants had differences in the FF metabolites between the small and large follicles, indicating that the FF at different developmental stages presents diverse metabolic profiles. We searched for metabolites in the FF and combined differential metabolites with embryonic development and clinical outcomes to analyze the correlations. These analyses showed that DHEA correlated negatively with the oocyte maturation rate and the high-quality embryo rate. This result could provide a reference for better pretreatment schemes before controlled ovarian hyperstimulation (COH), and could guide the optimization of culture systems for oocyte quality and embryo development. In addition, differential metabolites discovered in the FF could be used to predict clinical outcome.

## Conclusions

This present study found that the FF at different developmental stages has diverse metabolic characteristics. At the same time, the changes in the different metabolites in the FF would affect follicular development. DHEA, which is enriched in steroid hormone biosynthesis, correlated negatively with the oocyte maturation rate and the high-quality embryo rate, and thus could be used to estimate the predictive diagnostic potential of follicular development. Despite the limited sample size, these results provided us with a better understanding of the content of the FF, as well as the effects of different metabolites on follicular developmental competence. Further studies should be undertaken to validate the results with larger sample populations and populations at different states.

## Supplementary information

**Additional file 1: Supplemental Fig. 1** a Plots of PCA score in ESI positive and negative ion modes. b OPLS-DA score plot in the ESI positive ion mode. The OPLS-DA shows a predictive power (Q2) of 0.655 and a correlation index (R2Y) of 0.924, according to cross-validation. c OPLS-DA score plot in the ESI negative ion model. The OPLS-DA shows a prediction power (Q2) of 0.0921 and a correlation index (R2Y) of 0.469, according to cross-validation. d Volcano plots presenting the statistical significance of metabolites in the FF samples in the ESI positive ion mode. e Volcano plots presenting the statistical significance of metabolites in the FF samples in the ESI negative ion mode. f Change trends of 18 differential metabolites in the ESI positive ion mode. g Change trends of 12 differential metabolites in the ESI negative ion mode

**Additional file 2: Supplemental Fig. 2** a Heatmap plot of upregulation and downregulation for 18 differential metabolites with VIP > 1 and *P* < 0.05 identified in the ESI positive ion mode. b Heatmap plot of upregulation and downregulation for 12 differential metabolites with VIP > 1 and P < 0.05 identified in the ESI negative ion mode. c Overview of pathway analysis based on altered metabolisms suggested by MetPA in the ESI positive ion mode. d Overview of pathway analysis based on altered metabolisms suggested by MetPA in the ESI negative ion mode

**Additional file 3: Supplemental Table 1** Clinical epidemiological information of participants whose male spouses.

## Data Availability

The datasets used and/or analyzed during the current study are available from the corresponding author on reasonable request.

## Abbreviations

AFC: Antral follicle count
AMH: Anti-Müllerian Hormone
AUC: Area under the curve
BMI: Body mass index
COH: Controlled ovarian hyperstimulation
DOC: Deoxycorticosterone
DHEA: Dehydroepiandrosterone
E2: Estradiol
FF: Follicular fluid
FSH: Follicle-stimulating hormone
Gn: Gonadotropin
GnRH-a: Gonadotropin-releasing hormone agonist
GSH: Glutathione
HCG: Human chorionic gonadotropin
ICSI: Intracytoplasmic sperm injection
IVF: In vitro fertilization
KEGG: Kyoto encyclopedia of genes and genomes
LC-MS/MS: Liquid chromatography-tandem mass spectrometry
LH: Luteinizing hormone
OPLS-DA: Orthogonal partial least squares discriminant analysis
PCA: Principal component analysis
PCOS: Polycystic ovary syndrome
QC: Quality control
ROC: Receiver operator characteristic
SEM: Standard error of the mean
