# Supplementary figures and images for: Human follicular fluid shows diverse metabolic profiles at different follicle developmental stages
Source: Reprod Biol Endocrinol. 2020 Jul 23;18:74. doi: 10.1186/s12958-020-00631-x (PMC7376676; doi:10.1186/s12958-020-00631-x)

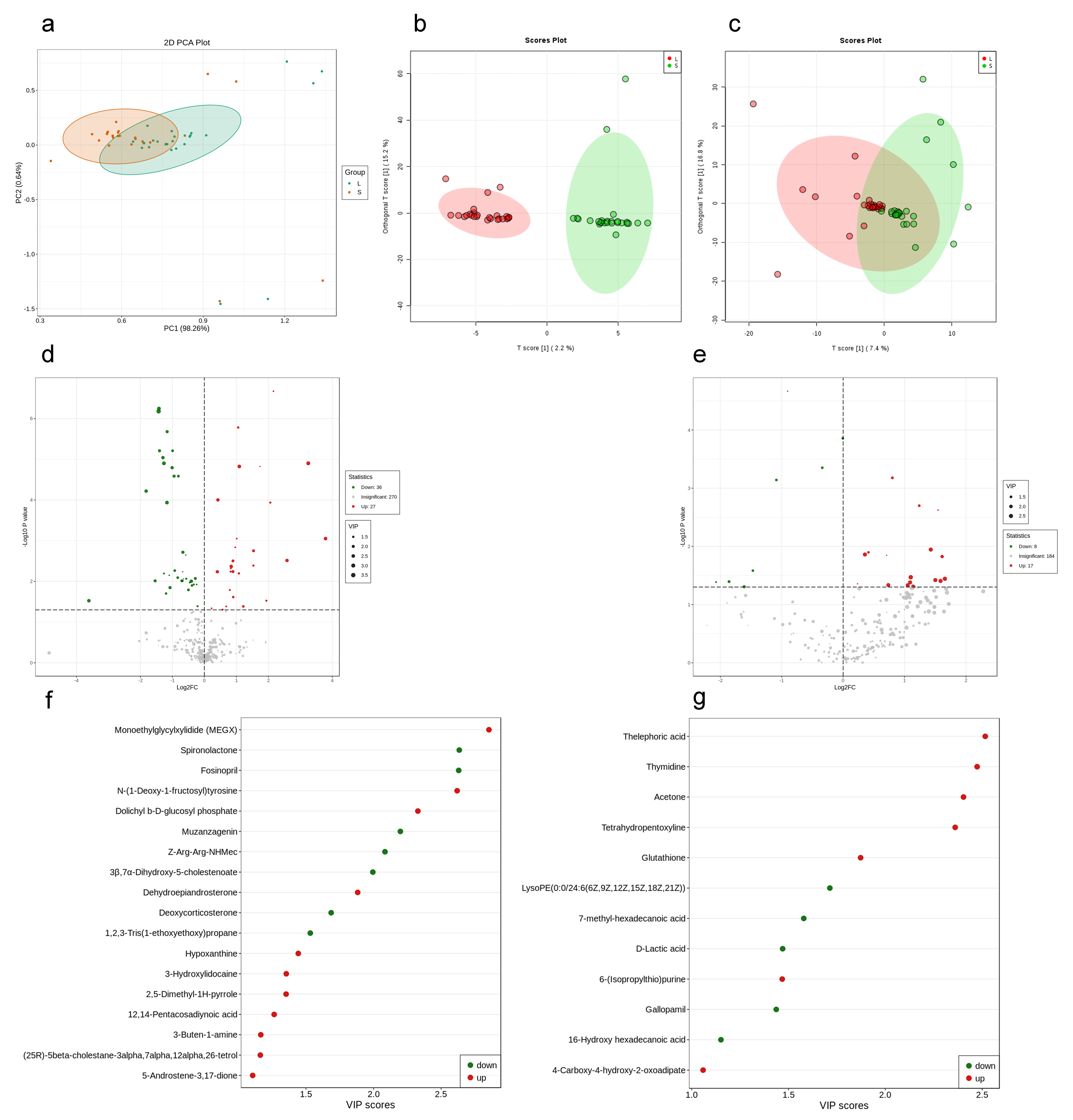

Supplement: Supplementary file 1 — Additional file 1: Supplemental Fig. 1 a Plots of PCA score in ESI positive and negative ion modes. b OPLS-DA score plot in the ESI positive ion mode. The OPLS-DA shows a predictive power (Q2) of 0.655 and a correlation index (R2Y) of 0.924, according to cross-validation. c OPLS-DA score plot in the ESI negative ion model. The OPLS-DA shows a prediction power (Q2) of 0.0921 and a correlation index (R2Y) of 0.469, according to cross-validation. d Volcano plots presenting the statistical significance of metabolites in the FF samples in the ESI positive ion mode. e Volcano plots presenting the statistical significance of metabolites in the FF samples in the ESI negative ion mode. f Change trends of 18 differential metabolites in the ESI positive ion mode. g Change trends of 12 differential metabolites in the ESI negative ion mode [file 12958_2020_631_MOESM1_ESM.tif]

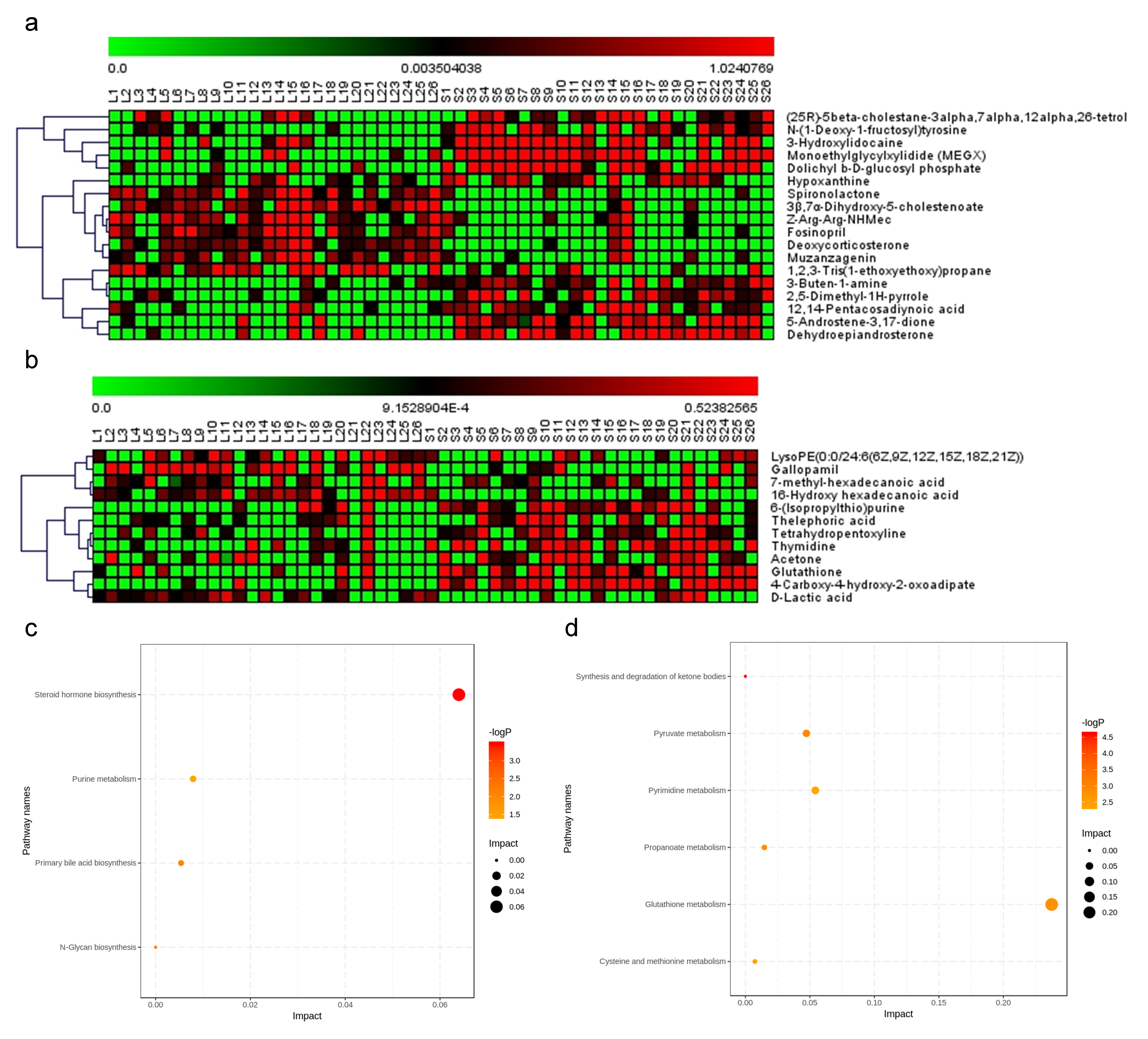

Supplement: Supplementary file 2 — Additional file 2: Supplemental Fig. 2 a Heatmap plot of upregulation and downregulation for 18 differential metabolites with VIP > 1 and P < 0.05 identified in the ESI positive ion mode. b Heatmap plot of upregulation and downregulation for 12 differential metabolites with VIP > 1 and P < 0.05 identified in the ESI negative ion mode. c Overview of pathway analysis based on altered metabolisms suggested by MetPA in the ESI positive ion mode. d Overview of pathway analysis based on altered metabolisms suggested by MetPA in the ESI negative ion mode [file 12958_2020_631_MOESM2_ESM.tif]
