# Supplementary material for: Human follicular fluid shows diverse metabolic profiles at different follicle developmental stages
Source: Reprod Biol Endocrinol. 2020 Jul 23;18:74. doi: 10.1186/s12958-020-00631-x (PMC7376676; doi:10.1186/s12958-020-00631-x)
Supplement: Supplementary file 3 — Additional file 3: Supplemental Table 1 Clinical epidemiological information of participants whose male spouses. [file 12958_2020_631_MOESM3_ESM.docx]

**Supplemental Table 1** Clinical epidemiological information of participants whose male spouses

| Parameters | Values |
| --- | --- |
| Age (year) | 30.83 ± 0.74 |
| BMI | 24.71 ± 0.58 |
| Semen volume (ml) | 3.84 ± 0.33 |
| Sperm Density (* 10^6^/ml) | 144.11 ± 22.53 |
| Sperm motility (%) | 80.83 ± 0.02 |
| Sperm deformity (%) | 83.08 ± 0.68 |
| Sperm fragmentation rate (%) | 15.83 ± 0.33 |

Data are presented as mean values and standard error.
